# Utilization of Matrix Metalloproteinase-9 Point-of-Care Immunoassay for Meibomian Gland Dysfunction Evaluation in Glaucoma Patients

**DOI:** 10.3390/jcm15072781

**Published:** 2026-04-07

**Authors:** Seung Hun Lee, Jin Hwan Park, Sung Chul Park, Si Hyung Lee

**Affiliations:** 1 Department of Ophthalmology, Soonchunhyang University Hospital Bucheon, Bucheon 14584, Republic of Korea; warring0207@naver.com (S.H.L.); 130504@schmc.ac.kr (J.H.P.); 138538@schmc.ac.kr (S.C.P.); 2 Department of Ophthalmology, College of Medicine, Soonchunhyang University, Cheonan 31538, Republic of Korea

**Keywords:** matrix metalloproteinase-9, meibomian gland dysfunction, glaucoma, ocular surface dysfunction

## Abstract

**Background/Objectives**: To evaluate the relationships between meibomian gland dysfunction (MGD), ocular surface parameters, and matrix metalloproteinase-9 (MMP-9)-mediated inflammation in glaucoma patients, we specifically assessed the impact of prostaglandin analogue use, preservative exposure, and number of medications. **Methods**: This retrospective cross-sectional study included patients treated with topical antiglaucoma medications for at least six months. Meibomian gland expressibility, meibum quality, and MGD grade were assessed along with tear film break-up time (TBUT), Schirmer I test, and Oxford staining score. Tear MMP-9 levels were measured using a Point-of-Care immunoassay (InflammaDry^®^) and graded on a 0 to 4 scale. **Results**: Elevated MMP-9 grades were significantly correlated with worsening meibum expressibility, meibum quality, and MGD grade (all *p* < 0.001), whereas no significant associations were found with traditional parameters such as TBUT and Schirmer I test. Prostaglandin analogue use was associated with worse meibomian gland parameters and higher MMP-9 levels compared to non-use. Patients receiving preservative-containing medications exhibited poorer meibomian gland parameters and MMP-9 levels, as well as worse corneal staining scores. An increased number of medications was associated with a stepwise deterioration in meibomian gland function and elevated MMP-9 levels. **Conclusions**: Prostaglandin analogue use, preservative exposure, and increased number of medications are significant factors associated with the exacerbation of MGD and ocular surface inflammation. Semi-quantitative grading of tear MMP-9 revealed a stepwise association with meibomian gland dysfunction severity that was not detected by conventional dry eye metrics, indicating that MMP-9 may be considered a potential indicator of subclinical ocular surface inflammation in glaucoma patients.

## 1. Introduction

Meibomian gland dysfunction (MGD) is a prevalent ocular condition that significantly impacts ocular comfort and visual health. It is characterized by impaired meibomian gland function, leading to inadequate secretion of specific lipids—such as wax esters and sterol esters—that are essential for maintaining the structural integrity of the tear film and preventing excessive evaporation [1,2]. Beyond lipid production, recent evidence has revealed that the meibomian glands are also involved in the secretion of key molecular signaling proteins, notably ectodysplasin A (EDA) [3]. EDA plays a crucial role in maintaining ocular surface homeostasis by promoting corneal epithelial cell proliferation and barrier function, and its levels are significantly diminished in MGD [4]. This dysfunction ultimately results in tear film instability, hyperosmolarity, and dry eye symptoms [5]. This dysfunction is especially relevant for patients with glaucoma, a progressive optic neuropathy often treated with topical medications, including prostaglandin analogues [6].

Prostaglandin analogues are effective in lowering intraocular pressure (IOP) but can exacerbate MGD by altering tear film dynamics and increasing ocular surface irritation [6]. For patients already experiencing MGD, the use of these medications may further compromise tear film integrity, resulting in a cycle of discomfort and decreased quality of life [7]. Furthermore, the inflammatory mediators associated with MGD can be intensified by the effects of prostaglandin therapy, complicating the management of both conditions [8,9].

Another critical factor is the presence of preservatives, such as benzalkonium chloride (BAK) and polyquad (PQ), in many glaucoma medications. These preservatives can induce ocular surface toxicity, leading to inflammation and disruption of meibomian gland function, thus worsening MGD symptoms and potentially reducing the efficacy of glaucoma treatment [10,11].

Given this multifactorial etiology, evaluating ocular surface inflammation is crucial for optimizing glaucoma management. Matrix metalloproteinase-9 (MMP-9), a pro-inflammatory marker which is elevated in dry eye disease, has emerged as one of key indicators of ocular surface inflammation. The MMP-9 Point-of-Care Immunoassay (InflammaDry^®^) allows for rapid assessment of this inflammatory activity in a clinical setting. The clinical utility of this assay stems from the fundamental role of MMP-9 in the vicious cycle of ocular surface disease. In the context of MGD, the reduced secretion of meibum and proteins leads to tear film instability and subsequent hyperosmolar stress. This stress triggers the corneal and conjunctival epithelia—the primary sources of MMP-9 on the ocular surface—to produce and release this enzyme. Elevated MMP-9 then compromises the ocular surface further by cleaving epithelial basement membrane components and tight junction proteins, thereby disrupting the corneal barrier function [12]. This pathological significance has led to the widespread clinical adoption of MMP-9 testing as a diagnostic marker of dry eye disease [13]. MMP-9-mediated inflammation has also been shown to contribute to chronic eyelid margin inflammation and vascular remodeling in MGD [14], which may further disrupt meibomian gland structure and function. As a result, MGD and evaporative dry eye may be reinforced in a self-perpetuating cycle. However, while MMP-9 is widely utilized in general dry eye assessment and its elevated expression in glaucoma patients has been previously reported [15], its specific correlation with meibomian gland changes in glaucoma patients remains less explored.

Therefore, this study aims to investigate the association between tear MMP-9 levels and meibomian gland parameters in glaucoma patients. By employing a quantitative grading system for MMP-9, rather than a binary assessment, we sought to provide a more nuanced understanding of how inflammatory intensity correlates with the structural and functional severity of MGD. Furthermore, we performed additional analyses to evaluate the effects of prostaglandin analogue (PG) use and preservative exposure as distinct pharmacologic factors, alongside cumulative medication burden, to better delineate their respective contributions to MMP-9-associated ocular surface inflammation in glaucoma patients.

## 2. Materials and Methods

This retrospective cross-sectional study was approved by the Institutional Review Board of Soonchunhyang University Hospital (No. SCHBC 2024-02-005) and was conducted in accordance with the tenets of the Declaration of Helsinki. The requirement for informed consent was waived due to the retrospective nature of the study.

### 2.1. Study Population and Patient Characteristics

**Figure 1:**
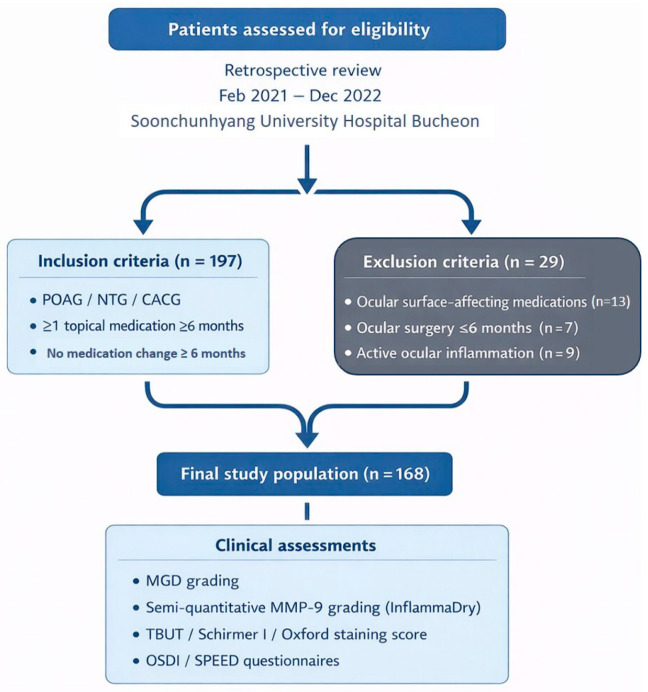
Flowchart of patient selection and experimental protocol. POAG; primary open angle glaucoma, NTG; normal tension glaucoma, CACG; chronic angle closure glaucoma, MGD; meibomian gland dysfunction, MMP-9; matrix metalloproteinases-9, TBUT; tear break-up time, OSDI; ocular surface disease index, SPEED; standard patient evaluation of eye dryness.

Medical records of patients who visited Soonchunhyang University Hospital Bucheon for regular glaucoma follow-up between February 2021 and December 2022 were retrospectively reviewed. Patients were eligible if they had a clinical diagnosis of primary open-angle glaucoma, normal-tension glaucoma, or chronic angle-closure glaucoma and had been treated with at least one topical antiglaucoma medication for 6 months or longer without any changes to their medication regimen for at least 6 months prior to enrollment. In this cohort, the indication for initiating or escalating topical glaucoma therapy was documented in the medical record as insufficient IOP control relative to an individualized target IOP, progressive structural or functional glaucomatous damage, or both, according to the treating glaucoma specialist, and treatment decisions were not based on meibomian gland status or ocular surface disease findings. Prostaglandin analogues were typically prescribed as first-line therapy in patients without contraindications, whereas additional agents (beta-blockers, carbonic anhydrase inhibitors, alpha-agonists, and fixed combinations) were added stepwise when IOP remained above target or when disease progression was observed. Patients were excluded if they were using systemic or topical medications known to affect ocular surface parameters or meibomian gland function (e.g., topical steroids or immunosuppressants), had undergone ocular surgery within the previous 6 months, or had active inflammatory ocular diseases such as uveitis. In addition, demographic and treatment-related data were collected from the medical records, including age, sex, glaucoma subtype, and the number and types of topical antiglaucoma medications used at the study visit. For each eligible patient, the following assessments were performed: (1) MGD grading, (2) tear MMP-9 measurement using InflammaDry, (3) evaluation of ocular surface parameters including tear film break-up time (TBUT), Schirmer I test, and Oxford staining score, and (4) completion of the Ocular Surface Disease Index (OSDI) and Standard Patient Evaluation of Eye Dryness (SPEED) questionnaires. The detailed patient selection process and experimental workflow are illustrated in Figure 1.

### 2.2. MGD Grading

**Figure 2:**
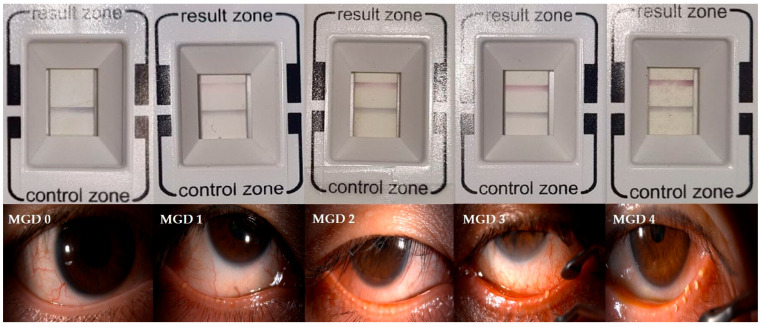
Representative images of InflammaDry^®^ results and meibomian gland dysfunction (MGD) grading. The top panel displays representative results of InflammaDry^®^ according to the 5-level grading scale, ranging from Grade 0 (negative) and Grade 1 (trace) to Grade 2 (weak positive), Grade 3 (moderate positive), and Grade 4 (strong positive). The bottom panel provides representative slit-lamp photographs illustrating the clinical severity of meibomian gland dysfunction, categorized as MGD grade 0 to 4.

The meibomian gland expressibility was evaluated using the meibomian forceps by applying moderate pressure to five glands on the lower eyelid, and the number of glands with secretion was examined. A score of 0 was assigned when all five glands expressed secretion, 1 when 3–4 glands expressed, 2 when 1–2 glands expressed, and 3 when no gland expressed secretion. The meibum quality was assessed by applying moderate pressure to eight central glands on the lower eyelid. Each gland was graded on a scale from 0 to 3 according to the quality of expressed meibum (0; clear meibum, 1; cloudy meibum, 2; cloudy meibum with granular debris, 3; toothpaste-like meibum). The scores of the eight glands were summed to obtain a total score ranging from 0 to 24. Because no universally accepted integrated staging system exists for MGD, overall MGD severity (Grade 0–4) was determined by integrating meibum quality, gland expressibility, and symptom profile according to the staging system proposed by the International Workshop on Meibomian Gland Dysfunction [16]. Representative slit-lamp photographs for each MGD grade are presented in Figure 2.

### 2.3. Measurement of MMP-9 Level Using the InflammaDry^®^ Assay

Tear MMP-9 levels were measured using the InflammaDry^®^ point-of-care immunoassay (RPS Diagnostics Inc., Sarasota, FL, USA) which detects elevated MMP-9 concentrations (>40 ng/mL). The test was performed in accordance with the manufacturer’s protocol. No topical medication was instilled for at least two hours prior to sampling. The sampling fleece was gently dabbed onto the lower palpebral conjunctiva approximately eight to ten times, allowing intermittent blinking between touches to minimize reflex tearing. The sampling tip was then immersed in the buffer vial for 20 s and placed horizontally for 10 min until the result window developed. The results were semi-quantitatively graded from 0 to 4 based on the intensity of the red band observed in the result window (Figure 1). This 5-grade scale has been clinically validated to show excellent diagnostic agreement with automated digital quantification, confirming that visual assessment by trained observers reliably reflects the biomarker concentration [17]. To minimize inter-observer variability, two independent examiners (Seung Hun Lee and J.H.P.) masked to the patients’ clinical data evaluated the band intensity. Discrepancies were resolved by consensus with a third senior examiner (Si Hyung Lee). This manual grading approach was utilized considering the practical challenges of performing standardized digital analysis for every test in a real-world clinical setting [15].

### 2.4. Evaluation of Ocular Surface Parameters (TBUT, Oxford Staining Score, Schirmer I Test)

After 10 min of measuring MMP-9 level, ocular surface parameters were evaluated, including TBUT, Oxford staining score and Schirmer I test. For TBUT, a fluorescein strip (Haag-Streit AG, Köniz, Switzerland) was gently applied to the inferior palpebral conjunctiva. Subjects were asked to blink naturally several times and the interval between the last complete blink and the first appearance of a dry spot on the corneal surface was recorded under slit lamp examination. The degree of epithelial damage on the cornea and conjunctiva was evaluated using the Oxford grading scale (Grade 0–4) [18]. Schirmer I test was performed without topical anesthesia using a sterile strip (Color Bar™, EagleVision Inc., Memphis, TN, USA) placed at the temporal one-third of the lower eyelid margin. Participants were instructed to close their eyes gently for 5 min, after which the length of the moistened area was measured in millimeters.

### 2.5. SPEED and OSDI Questionnaires

All participants were asked to complete the SPEED and OSDI questionnaires. Both questionnaires are simple and time-efficient tools for assessing the severity of dry eye symptoms in patients [19,20]. The SPEED questionnaire evaluates both the frequency and intensity of dry eye related symptoms. It consists of items addressing the onset, frequency, and severity of ocular discomfort, yielding a total score ranging from 0 to 28, with higher scores indicating more severe symptoms. The OSDI questionnaire comprises 12 items divided into three subscales—ocular symptoms, vision-related function, and environmental triggers—and produces a composite score from 0 to 100, where higher scores reflect greater symptom severity.

### 2.6. Statistical Analysis

All statistical analyses were performed using IBM SPSS Statistics version 21.0 (IBM Corp., Armonk, NY, USA). The associations between MMP-9 levels and MGD grade, ocular surface parameters, OSDI, and SPEED scores were examined using Spearman’s rank correlation test. Comparisons between groups, according to PG use and preservative status, were conducted using the Mann–Whitney U test. Differences based on the number of topical glaucoma medications were analyzed using the Kruskal–Wallis test, followed by Mann–Whitney U tests with Bonferroni correction for post hoc pairwise comparisons. A *p*-value < 0.05 was considered statistically significant.

## 3. Results

### 3.1. Baseline Characteristics of Study Population

A total of 168 patients were included in the analysis. The mean age was 59.43 ± 11.18 years. Mean MGD and MMP-9 grades were 2.64 ± 1.13 and 1.96 ± 1.24, respectively (range for both, 0–4). Mean TBUT, Schirmer I test, and Oxford staining scores were 3.59 ± 2.04, 11.27 ± 8.24, and 1.60 ± 0.80. The mean OSDI and SPEED scores were 22.63 ± 18.37 and 6.24 ± 4.05 (Table 1).

### 3.2. Associations Between MMP-9 Grade and Meibomian Gland/Ocular Surface Parameters

The associations between MMP-9 grade and ocular surface parameters are summarized in Table 2. Notably, elevated MMP-9 grades were strongly associated with worsening meibomian gland function, both meibum expressibility (r = 0.413, *p* < 0.001) and meibum quality (r = 0.436, *p* < 0.001). Consequently, the overall MGD grade showed a significant positive correlation with the MMP-9 grade (r = 0.441, *p* < 0.001), indicating that higher levels of ocular surface inflammation may be closely linked to structural and functional meibomian gland compromise. In contrast, traditional ocular surface metrics did not show significant correlations with MMP-9 levels. Neither TBUT (r = −0.024, *p* = 0.757) nor Schirmer I test values (r = −0.029, *p* = 0.714) were significantly associated with MMP-9 grade. Similarly, the Oxford staining score (*p* = 0.358) and subjective symptom scores (OSDI and SPEED) did not exhibit a statistically significant correlation with MMP-9 positivity. These findings suggest that MMP-9 elevation may reflect inflammatory changes related to meibomian gland dysfunction, which may occur independently of changes in aqueous tear production or clinically visible epithelial damage.

### 3.3. Associations Between Prostaglandin Analogue Use and Meibomian Gland/Ocular Surface Parameters

Differences according to PG use were then analyzed (Table 3). Compared with the non-PG group, the PG group showed significantly poorer meibum expressibility (*p* = 0.017) and meibum quality (*p* = 0.001), and a higher MGD grade (*p* = 0.006). The MMP-9 grade was also significantly higher in the PG group (*p* < 0.001). No significant differences were observed in TBUT, Schirmer I test, Oxford staining score, or OSDI score between the two groups, whereas the SPEED score was significantly higher in the PG group (*p* = 0.033). Because preservatives such as BAK and PQ may influence ocular surface findings, a subgroup comparison was performed between preservative-free prostaglandin users (PG-PF, *n* = 42) and preservative-free non-prostaglandin users (non-PG-PF, *n* = 24). The PG-PF group showed significantly poorer meibum expressibility (*p* = 0.035), worse meibum quality (*p* = 0.002), and a higher MGD grade (*p* = 0.022), as well as a higher MMP-9 grade (*p* = 0.003). As in the primary analysis, no significant differences were observed in TBUT, Schirmer I test, Oxford staining score, or OSDI score, while the SPEED score remained significantly higher in the PG-PF group (*p* = 0.029).

### 3.4. Associations Between Preservative Use and Meibomian Gland/Ocular Surface Parameters

Associations between preservative use and ocular surface parameters were further examined (Table 4). Patients using preservative-containing medications had significantly worse meibum expressibility and quality (both *p* < 0.001), and a higher MGD grade (*p* < 0.001), compared with preservative-free users. The preservative group also demonstrated a higher MMP-9 grade (*p* = 0.009) and worse Oxford staining scores (*p* = 0.004). No significant differences were noted in TBUT or Schirmer I test. OSDI scores were higher in the preservative group (*p* = 0.034), whereas SPEED scores did not differ significantly.

### 3.5. Associations Between Number of Glaucoma Medications and Meibomian Gland/Ocular Surface Parameters

Finally, differences according to the number of glaucoma medications were analyzed (Table 5). An increasing number of medications were associated with progressively poorer meibum expressibility (*p* = 0.010), worse meibum quality (*p* = 0.003), and a higher MGD grade (*p* = 0.016). MMP-9 grade also increased significantly with the number of medications (*p* = 0.049). Oxford staining scores differed significantly among the groups (*p* = 0.026). No significant differences were found in TBUT, Schirmer I test, OSDI score, or SPEED score according to medication number.

## 4. Discussion

In this study, we investigated the associations between MGD, ocular surface parameters, and MMP-9-based inflammatory status in glaucoma patients treated with topical medications. Our findings demonstrated a consistent relationship between elevated MMP-9 grades and impaired meibomian gland function, with meibum expressibility, meibum quality, and overall MGD grade showing moderate positive correlations with MMP-9 level. Notably, this study is the first to comprehensively analyze these inflammatory and structural changes with specific stratification by prostaglandin analogue use and the presence of preservatives, including preservative-free formulations. These results support previous reports indicating that MMP-mediated inflammation plays an important role in the pathophysiology of MGD and evaporative dry eye disease [16,21,22]. In contrast, we observed no significant association between MMP-9 levels and conventional tear film metrics such as TBUT and Schirmer I test. This discrepancy suggests that MMP-9 elevation may be considered a more sensitive indicator of active ocular surface inflammation and meibomian gland dysfunction compared to traditional clinical tests, particularly in detecting subclinical ocular surface inflammation in glaucoma patients.

The use of PG eyedrops was associated with significantly worse meibomian gland parameters and higher MMP-9 grades compared with non-users. Our findings align with prior studies reporting that prostaglandin analogues can induce or exacerbate meibomian gland dysfunction [6,9,23]. Of particular interest in our study is that this deleterious effect persisted in the preservative-free PG group compared to the preservative-free non-PG group. This raises the possibility that the prostaglandin molecule itself may have pro-inflammatory or meibotoxic properties, as previously reported [6,9], independent of BAK toxicity. While preservatives are established aggravators of the ocular surface [24,25], our data highlights the need for vigilance regarding MGD exacerbation even in patients prescribed preservative-free PG formulations. Furthermore, these results support the potential clinical utility of the MMP-9 immunoassay as an adjunctive tool for evaluating MGD status in patients treated with PG eyedrops.

Preservative exposure was also significantly associated with worse meibomian gland function and higher inflammatory burden. Patients receiving preservative-containing antiglaucoma medications exhibited significantly poorer meibum expressibility and quality, higher MGD grades, and elevated MMP-9 levels compared with those using preservative-free formulations. These findings are consistent with the well-established cytotoxic and pro-inflammatory effects of preservatives on the ocular surface, including epithelial disruption, goblet cell loss, and tear film instability [24,25]. Notably, TBUT and Schirmer I test did not differ significantly between the preservative-containing and preservative-free groups, further suggesting that these traditional metrics may be insufficient to detect subclinical change identified by measuring MMP-9.

The number of topical glaucoma medications also demonstrated a dose-dependent relationship with ocular surface damage. Patients using a higher number of medications exhibited stepwise worsening of meibomian gland parameters, increased MGD grades, and elevated MMP-9 levels. This trend is likely attributable to the cumulative toxicity from preservatives and the increased frequency of daily instillations, which collectively amplify corneal epithelial toxicity and inflammatory activity. Consistent with previous reports indicating that ocular surface disease prevalence rises to 60–70% in patients using three or more agents [26], our findings similarly demonstrated a stepwise association between medication burden and ocular surface inflammation parameters.

Recent studies have also highlighted the relevance of MMP-9–mediated inflammation in glaucoma patients. Elevated tear MMP-9 levels have been associated with increased tear film osmolarity, reduced TBUT, lower Schirmer’s test values and worse corneal and conjunctival staining scores, reflecting more severe dry eye disease [27,28]. Although the MMP-9 point-of-care immunoassay (InflammaDry) has been widely used in the evaluation of dry eye disease, relatively few studies have applied this tool specifically to glaucoma populations. Among the available literature, Kim et al. demonstrated that patients with primary open-angle glaucoma exhibit a higher rate of MMP-9 overexpression compared with healthy controls [15]. Similarly, Zaleska-Żmijewska et al. reported that patients treated with BAK-containing prostaglandin analogues exhibited higher MMP-9 positivity than those receiving preservative-free medications, underscoring the role of preservative exposure in ocular surface inflammation [28]. Our study extends these findings by utilizing a semi-quantitative grading system for MMP-9, rather than a simple binary assessment, to demonstrate a stepwise correlation with the structural and functional severity of MGD. Furthermore, by specifically differentiating the impacts of prostaglandin analogues, preservative exposure, and the cumulative medication burden, we identified that both the chemical properties of the drug and the overall treatment regimen may contribute to the severity of ocular surface inflammation. Early identification and stratification of patients with elevated MMP-9 may therefore enable clinicians to initiate timely, individualized interventions—such as anti-inflammatory therapy, lipid-enhancing artificial tears, warm compresses, or transitioning to preservative-free formulations—to prevent further deterioration. These strategies may improve patient comfort, promote better adherence, and ultimately contribute to more stable intraocular pressure control.

This study has several limitations. First, due to its retrospective design, patients with pre-existing ocular surface disease or MGD may have been inadvertently included, making it difficult to determine whether the observed ocular surface changes were caused by glaucoma medications or were present beforehand. In particular, the possibility that patients with severe MGD may have been prescribed preservative-containing drops due to their chronic, long-term condition cannot be entirely ruled out. A larger, prospective study involving treatment-naïve glaucoma patients and appropriate control groups would therefore be necessary to clarify the causal relationship between medication exposure, MGD severity, and MMP-9 expression. Second, although a semi-quantitative grading system was used to interpret InflammaDry results, variability between interpreters may still occur, potentially affecting the precision of MMP-9 assessment. Likewise, MGD severity was evaluated without the use of meibography, which could have provided complementary structural information regarding meibomian gland dropout. Furthermore, more objective and automated diagnostic tools, such as non-invasive tear break-up time (NIBUT) and tear osmolarity measurement, were not utilized in this study. Incorporating these quantitative metrics in future research would allow for a more precise and comprehensive analysis of the inflammatory and functional changes on the ocular surface. Despite these limitations, the strengths of this study include a relatively large sample size and a comprehensive evaluation of both inflammatory and functional ocular surface parameters using semi-quantitative MMP-9 grading.

## 5. Conclusions

In conclusion, our findings demonstrated that prostaglandin analogue use, preservative exposure, and increased medication burden were each associated with worsening meibomian gland dysfunction and ocular surface inflammation in glaucoma patients. The strong correlation observed between MMP-9 levels and structural meibomian gland changes—in the absence of similar findings with conventional metrics—supports the potential clinical utility of inflammatory biomarker testing. Incorporating semi-quantitative MMP-9 assessment into routine glaucoma evaluation may allow clinicians to identify MGD-driven ocular surface inflammation before the development of overt tear film instability or epithelial damage, thereby enabling earlier, mechanism-based interventions.

## Figures and Tables

**Table 1:** Baseline characteristic of study participants (*n* = 168).

Variables	Values
Total participants, *n*	168
Age (years)	59.43 ± 11.18 (range: 24−80)
Sex	
M (%)	77 (45.8)
F (%)	91 (54.2)
Meibum expressibility	1.82 ± 0.90 (range: 0–3)
Meibum quality	10.18 ± 6.16 (range: 0–24)
MGD grade	2.64 ± 1.13 (range: 0–4)
MMP-9	
Negative [Grade 0] (%)	26 (15.5)
Trace [Grade 1] (%)	35 (20.8)
Weak positive [Grade 2] (%)	45 (26.8)
Moderate positive [Grade 3] (%)	43 (25.6)
Strong positive [Grade 4] (%)	19 (11.3)
Tear break up time (s)	3.59 ± 2.04
Schirmer I test (mm)	11.27 ± 8.24
Oxford staining score	1.60 ± 0.80
OSDI (scores)	22.63 ± 18.37
SPEED (scores)	6.24 ± 4.05

Values presented as mean ± standard deviation or number (%), MGD; meibomian gland dysfunction, MMP-9; matrix metalloproteinases-9, OSDI; ocular surface disease index, SPEED; standard patient evaluation of eye dryness.

**Table 2:** Associations between MMP-9 grade and meibomian gland/ocular surface parameters.

Variables	MMP-9 Grade					*R*	*p*-Value
	0 (*n* = 26)	1 (*n* = 35)	2 (*n* = 45)	3 (*n* = 43)	4 (*n* = 19)		
Age (years)	58.81 ± 13.01	55.83 ± 11.90	59.80 ± 9.39	61.84 ± 11.20	60.63 ± 10.29		0.201 *
Meibum expressibility	1.27 ± 0.87	1.49 ± 0.91	1.80 ± 0.89	2.19 ± 0.69	2.37 ± 0.68	0.413	<0.001 ^†^
Meibum quality	5.73 ± 4.95	8.57 ± 6.67	10.36 ± 5.85	12.42 ± 5.29	13.79 ± 5.10	0.436	<0.001 ^†^
MGD grade	1.77 ± 1.27	2.26 ± 1.19	2.67 ± 1.00	3.12 ± 0.79	3.37 ± 0.68	0.441	<0.001 ^†^
Tear break up time (s)	3.46 ± 2.70	3.77 ± 21.4	3.93 ± 1.81	3.35 ± 1.58	3.16 ± 2.29	−0.024	0.757 ^†^
Schirmer I test (mm)	9.92 ± 6.82	12.71 ± 9.67	11.62 ± 8.17	11.33 ± 8.36	9.53 ± 7.21	−0.029	0.714 ^†^
Oxford staining score	1.54 ± 0.81	1.51 ± 0.74	1.67 ± 0.73	1.47 ± 0.85	1.95 ± 0.91	0.071	0.358 ^†^
OSDI (scores)	18.58 ± 14.72	21.94 ± 18.28	20.11 ± 14.94	25.75 ± 20.21	28.33 ± 24.49	0.137	0.076 ^†^
SPEED (scores)	5.96 ± 4.55	5.91 ± 3.68	6.57 ± 3.75	6.16 ± 3.97	6.63 ± 5.03	0.046	0.554 ^†^

* Kruskal–Wallis test, ^†^ Spearman correlation, MMP-9; matrix metalloproteinases-9, OSDI; ocular surface disease index, SPEED; standard patient evaluation of eye dryness.

**Table 3:** Associations between prostaglandin analogue use and meibomian gland/ocular surface parameters.

Variables	PG (*n* = 122)	Non-PG (*n* = 46)	*p*-Value *	PG-PF (*n* = 42)	Non-PG-PF (*n* = 24)	*p*-Value *
Age (years)	59.11 ± 11.14	60.30 ± 11.35	0.300	60.29 ± 9.43	58.58 ± 11.63	0.931
Meibum expressibility	1.93 ± 0.84	1.52 ± 0.98	0.017	1.60 ± 0.82	1.08 ± 0.88	0.035
Meibum quality	11.18 ± 5.94	7.54 ± 6.01	0.001	8.71 ± 5.02	4.71 ± 4.25	0.002
MGD grade	2.82 ± 0.96	2.15 ± 1.38	0.006	2.43 ± 0.96	1.63 ± 1.31	0.022
MMP-9 grade	2.24 ± 1.11	1.24 ± 1.28	<0.001	2.02 ± 1.19	1.08 ± 1.06	0.003
Tear break up time (s)	3.45 ± 1.69	3.96 ± 2.75	0.927	3.21 ± 1.40	3.42 ± 2.10	0.736
Schirmer I test (mm)	11.51 ± 8.64	10.65 ± 7.13	0.820	11.14 ± 7.64	10.67 ± 7.02	0.778
Oxford staining score	1.61 ± 0.80	1.54 ± 0.80	0.515	1.40 ± 0.79	1.29 ± 0.55	0.629
OSDI (scores)	23.44 ± 18.58	20.46 ± 17.79	0.221	19.20 ± 17.73	18.89 ± 17.97	0.603
SPEED (scores)	6.61 ± 4.00	5.26 ± 4.04	0.033	6.61 ± 3.85	4.50 ± 2.79	0.029

* Mann–Whitney U test, PG; prostaglandin analogue, PF; preservative free, MGD; meibomian gland dysfunction, MMP-9; matrix metalloproteinases-9, OSDI; ocular surface disease index, SPEED; standard patient evaluation of eye dryness, PG-PF: prostaglandin analogue without preservatives.

**Table 4:** Associations between preservative use and meibomian gland/ocular surface parameters.

Variables	Preservative Use		*p*-Value *
	No (*n* = 93)	Yes (*n* = 75)	
Age (years)	59.37 ± 11.14	59.52 ± 11.30	0.916
Meibum expressibility	1.51 ± 0.88	2.20 ± 0.77	<0.001
Meibum quality	7.91 ± 5.55	13.00 ± 5.75	<0.001
MGD grade	2.24 ± 1.14	3.13 ± 0.90	<0.001
MMP-9 grade	1.74 ± 1.20	2.24 ± 1.24	0.009
Tear break up time (s)	3.99 ± 2.44	3.27 ± 1.59	0.100
Schirmer I test (mm)	10.95 ± 8.36	11.54 ± 8.18	0.200
Oxford staining score	1.43 ± 0.71	1.80 ± 0.87	0.004
OSDI (scores)	19.61 ± 16.03	26.38 ± 20.40	0.034
SPEED (scores)	5.90 ± 3.64	6.67 ± 4.50	0.326

* Mann- Whitney U test, MMP-9; matrix metalloproteinases-9, OSDI; ocular surface disease index, SPEED; standard patient evaluation of eye dryness.

**Table 5:** Associations between number of glaucoma medications and meibomian gland/ocular surface parameters.

Variables	Number of Glaucoma Medication (s)			*p*-Value *
	1 (*n* = 106)	2 (*n* = 51)	3 (*n* = 11)	
Age (years)	60.17 ± 10.46	58.47 ± 12.92	56.82 ± 9.11	0.376
Meibum expressibility	1.66 ± 0.91	2.04 ± 0.82	2.27 ± 0.78	0.010
Meibum quality	8.90 ± 5.68	11.92 ± 6.13	14.55 ± 7.29	0.003
MGD grade	2.44 ± 1.18	2.94 ± 0.94	3.09 ± 1.04	0.016
MMP-9 grade	1.79 ± 1.27	2.20 ± 1.20	2.55 ± 0.82	0.049
Tear break up time (s)	3.64 ± 2.13	3.47 ± 1.74	3.64 ± 2.54	0.952
Schirmer I test (mm)	11.03 ± 7.62	12.27 ± 9.53	9.00 ± 7.62	0.146
Oxford staining score	1.47 ± 0.80	1.84 ± 0.78	1.64 ± 0.67	0.026
OSDI (scores)	20.24 ± 17.17	26.67 ± 20.48	26.89 ± 16.57	0.052
SPEED (scores)	6.01 ± 3.96	6.75 ± 4.48	6.09 ± 2.46	0.657

* Kruskal–Wallis test, MGD: meibomian gland dysfunction, MMP-9; matrix metalloproteinase-9, OSDI; ocular surface disease index, SPEED; standard patient evaluation of eye dryness.

## Data Availability

The datasets generated and analyzed during the current study are available from the corresponding author on reasonable request.

## Abbreviations

The following abbreviations are used in this manuscript:

MGD	Meibomian Gland Dysfunction
MMP-9	Matrix Metalloproteinase-9
TBUT	Tear Break Up Time
PG	Prostaglandin analogue
BAK	Benzalkonium chloride
PQ	Polyquad
OSDI	Ocular Surface Disease Index
SPEED	Standard Patient Evaluation of Eye Dryness
